# Effect of neuroticism on risk of cardiovascular disease in depressed persons - a Swedish population-based cohort study

**DOI:** 10.1186/s12872-017-0604-4

**Published:** 2017-07-11

**Authors:** Aysha Almas, Jette Moller, Romaina Iqbal, Yvonne Forsell

**Affiliations:** 1 Department of Public Health Sciences, Karolinska Institutet, 171 77 Stockholm, Sweden; 2 Department of Medicine, Aga Khan University, Karachi, Pakistan; 3 Department of Community Health Sciences, Aga Khan University, Karachi, Pakistan

**Keywords:** Depression, Neuroticism, Cardiovascular diseases, Cohort study

## Abstract

**Background:**

The relationship between neuroticism, depression and cardiovascular disease (CVD) is complex and has so far not been studied in depth. The aim of this study was to determine if neuroticism is an effect-modifier in the association between depression and CVD. Data derived from a longitudinal cohort study on mental health, work and relations among adults (20–64 years), including 10,443 individuals. Depression was assessed using the Major Depression Inventory (MDI) and neuroticism by the Swedish Scale of Personality (SSP). Outcomes of cardiovascular disease were register-based from the National inpatient register.

**Results:**

Both depression (OR 1.9 (95%CI 1.4, 2.5)) and high levels of neuroticism (OR 1.2 (95%CI 1.1–1.3)) were associated with increased risk of CVD. The combined effect of depression and neuroticism on the risk of CVD revealed HRs ranging from 1.0 to 1.9 after adjusting for age and gender, socioeconomic position, prevalent hypertension and diabetes. Almost similar associations were seen after further adjustment for lifestyle factors.

**Conclusion:**

Neuroticism increased the risk of CVD in depressed persons. We found synergistic interaction between neuroticism and depression status in predicting future risk of CVD.

## Background

Personality traits signify the ability of a person to respond to the environment and this distinguishes one person from another. One of the personality traits; neuroticism is operationally defined by items referring to irritability, anger, sadness, anxiety, worry, hostility, self-consciousness, and vulnerability that have been found to be substantially correlated with one another [1, 2]. Persons with higher levels of neuroticism are often self-critical, sensitive to the criticism of others, and feel personally inadequate [3]. This personality trait appears to be correlated with a wide range of mental and physical health problems [4] including depression [5], stroke and increased coronary heart disease mortality [6, 7]. On the other hand depression increases the risk of cardiovascular diseases, such as heart disease, hypertension and stroke [8–12]. However, it is not yet known if high or low level of neuroticism modifies the relation between depression and CVD. Although in some scales the items that define neuroticism overlaps with symptoms of depression and anxiety which complicates the interpretation of correlations between these disorders [13, 14].

Persons with a high degree of neuroticism more frequently experience higher levels of psychosocial stress which in turn can lead to elevated blood pressure, atherosclerosis, and risky health behaviors such as poor diet, physical inactivity, smoking, sleep disturbances, or lower treatment adherence [15]. Associations with even more serious outcomes have been reported: the British Health and Lifestyle Survey concluded that neuroticism was associated with a higher mortality risk in coronary heart disease but not in stroke [16]. These findings that were later confirmed in a pooled analysis from three cohort studies [6]. In several studies attention has been paid to the association between neuroticism and depression after CVD events. For example, two studies of a post-stroke cohort, have suggested that pre-morbid neuroticism predicts post-stroke depression [17, 18]. However, to our knowledge, only one previous study has addressed the question of whether the effect of neuroticism on future risk of CVD differs in the presence of depression. This study, the Longitudinal Aging Study Amsterdam (LASA), reported that depression is only predictive for future stroke in the absence of high neuroticism [19]. The proposed hypothesis in LASA was that people with lower degree of neuroticism, had more vascular disease leading to incident cardiovascular disease. This implies that people with lower levels of neuroticism might have more atherosclerosis and hence more cardiovascular events. Additionally, the results from the LASA study were limited by the non-participation rate of participants with higher degree of neuroticism at baseline and a relatively low number of incident stroke. In the present study we use data from a longitudinal population-based Swedish cohort where depression has previously been shown to be associated with an increased risk for CVD (OR 1.9 (95%CI 1.4,2.5) [20].

The aim of this study was to investigate the effect of neuroticism on the association between depression and cardiovascular disease. We hypothesize that people with high levels of neuroticism who become depressed have a higher increased risk of CVD than those with low levels of neuroticism.

## Methods

The data originates from the PART study (acronym in Swedish for: Psykisk hälsa, Arbete och RelaTioner, In English: Physical Health, Work and Relations), a longitudinal study of mental health, work and relations among adults residing in Stockholm County, Sweden [21]. The study included three data collections, wave 1 (W1) in 1998–2000, wave 2 (W2) in 2001–2003 and wave 3 (W3) in 2010. In the present study we used data from W1 and W2. Data from W3 was not used as it was collected after 10 years of initial exposure (depression) and self-reported CVD outcomes were reported only for 50.5% (*n* = 5228) of the participants. In each wave participants responded to a postal questionnaire including questions on risk and protective factors for mental health as well as psychiatric rating scales. In the case of missing answers in the questionnaires, the persons were contacted by telephone. In the present study, all participants from W1 were followed up between 2001 to 2014 for occurrence of cardiovascular diseases through register data from the National Patient Register (NPR) during 2008–2011 [22]. The Ethical Review Board at Karolinska Institutet, Stockholm, approved the study (2010/1185–31/596:260, 01–218, 04–528/3 and 09–880) and written informed consent was obtained from all participants.

The PART study intended to include 19,744 randomly selected Swedish citizens of which 19,457 could be reached, and 10,443 individuals responded to the questionnaire at W1 (participation rate 53%). Non-response analyses have been executed using available administrative registers, and participation was associated with female gender, higher age, higher income and education, being born in the Nordic countries and having no previous psychiatric diagnosis in inpatient registers [23]. In the following two waves the participation rates were 83% (*n* = 8622) and 61% (*n* = 5228). Attrition in W2 was associated with similar factors as in W1 [24].

### Depression

Depression was evaluated using the Major Depression Inventory (MDI) based on responses given in W1 or W2. For those who were depressed in both waves, the highest score was used to assess level of severity. Severity of depression was based on the MDI score and categorized as follows: not depressed (MDI score < 20), mild depression [20–24], moderate [25–29] and severe depression (MDI >30) [25]. The Cronbach’s alpha coefficient for internal consistency for MDI in W1 was 0.91 and in W2 was 0.90. The MDI has shown high validity in both clinical and non-clinical samples, including the PART study [26–28] and can be used to make diagnoses according to DSM-IV.

### Neuroticism

The Swedish Universities’ Scales of Personality (SSP) was used for assessment of the personality trait neuroticism (see Appendix for detailed information) [29] and has previously been widely used specially in Swedish patient populations for assessment of personality [30, 31]. The SSP consists of 91 items grouped into 13 different scales. Each scale has seven items and each item is presented as a statement with a four-point response format, ranging from 1 to 4, 1 point for “disagree” and so on to 4 points for “agree”. Scale scores were calculated by summing up the scores from individual items and then dividing by the number of input items. T-scores were then computed from normative standard scores. T-scores are standardized scores on each dimension for each type and a score of 50 represents the mean [29]. The reported Cronbach’s alpha coefficient for internal consistency of SSP ranges from 0.59 to 0.84, and the mean inter-item correlations (MIIC) ranges from 0.17 to 0.43. Previous analyses on the data showed that the scale inter correlation matrix produced a three-factor solution using Principal axis factoring; factor 1 reflecting neuroticism; factor 2 aggressiveness; and factor 3 extraversion. Neuroticism was assessed using three scales from the SSP [29]: somatic trait anxiety, stress susceptibility and embitterment. No imputation was done since no partially missing data was available for the three subscales, i.e. somatic trait anxiety, stress susceptibility and for embitterment. Scores for neuroticism was divided into tertiles at 33.3 and 66.6; lowest tertile 1 ≤ 33.4, middle tertile between 33.5 to 66.6 and highest tertile >66.6. Lowest and middle tertiles were then merged to form the lower level neuroticism and highest tertile as high-level neuroticism. This categorization was used since we were more interested in those who scored higher on the neurotic personality scale. Low-level neuroticism was used as reference group in the analysis.

### Cardiovascular disease

Cardiovascular disease (CVD) was extracted from hospital discharge diagnoses in the National Patient Register (NPR) during 2001 to 2014 and data was linked to the participants through a unique Personal Identity Number (PIN). A previous validation of the NPR by the National Board of Health and Welfare showed that 85–95% of all diagnoses in the NPR are valid [32, 33]. The diagnosis which were followed were: ischemic/hypertensive heart disease; hypertensive diseases (ICD10 codes: I11–13), ischemic heart diseases (ICD10: I20–25), heart failure (ICD10: I50), other peripheral vascular diseases, embolism and thrombosis (ICD10: I73–74) and stroke (ICD10: I60–67 and I69). Participants reporting a history of ischemic/hypertensive heart disease or stroke at or before baseline *n* = 267 (2.6%) were excluded from further analysis.

### Covariates

Age, gender, socioeconomic position [34], prevalent ischemic heart disease, hypertension, stroke and diabetes were used in the analysis as covariates [35–37]. Smoking, hazardous alcohol use, body mass index (BMI) and physical activity were used as mediators since they are traditional risk factors for both depression and CVD [9, 38–42]. Four age categories were used: 30–45; 46–60; 61–70; and >71 years. SEP in W1 was measured using occupational groups defined according to the Nordic Standard Occupational Classification (NSOC) of 1989 [43] and classified into five groups: high/intermediate level salaried employees; assistant non-manual employees; skilled workers; unskilled workers; and self-employed (including farmers). Participants were considered as physically active if they reported exercising habitually at least three times a week. Smoking habits reported in W2 were classified as regular smoker, occasional smoker, previous smoker and never smoker [44] Hazardous alcohol use in W1 and W2 was assessed by the Alcohol Use Disorders Identification Test (AUDIT) [45] and dichotomized following the Swedish cut-off points (≥8 points for men and ≥6 points for women) [46]. If data was available from both W1 and W2, a combined variable was created for both waves indicating presence in both waves or maximum value in either wave for a continuous variable like BMI.

### Statistical analyses

Descriptive analyses were performed to report mean and standard deviation (SD) for continuous variables and frequency and percentage for categorical variables. Imputation of missing values for MDI was accomplished by the mean value of the questions in the answered items when there were missing answers for one or two of the ten questions. If answers were missing for three or more questions; 0.2% (*n* = 23) in W1 and 1.4% (*n* = 121) in W2, the response was left as missing. The maximum number of missing values for questions were as follows; somatic trait anxiety 0.3% (*n* = 29), stress susceptibility 0.3% (*n* = 29) and for embitterment 0.4% (*n* = 29). As there was no partially missing data on the SSP, no imputation was done for the three subscales i.e. somatic trait anxiety, stress susceptibility and embitterment. Multicollinearity was checked between MDI and SSP and was not significant (Variation Inflation Factor = 1).

Multiplicative and Additive interaction were estimated. Multiplicative interaction was estimated using main effect model with and without multiplicative interaction between depression and neuroticism and *p*-value was calculated using the Loglikelihood test [47]. Additive interaction was estimated measuring interaction on an additive scale as it is appropriate for assessing the public health importance of interactions [48] and based on dummy variables of depression and levels of neuroticism. Four dummy variables were created; low level neuroticism with no depression (reference), low level neuroticism with depression, high level neuroticism with no depression, and high level neuroticism with depression. Synergy index (S) was calculated to confirm if additive interaction was present. S = 1 means no interaction or exactly additivity; S > 1means positive interaction or more than additivity; S < 1means negative interaction or less than additivity. S can range from 0 to infinity [49]. Logistic regression was used to calculate OR where required.

Cox regression models were constructed to determine hazard ratios with 95% confidence intervals using survival analysis. Participants were followed from January 2001 to December 2014 and endpoints considered were: time of IHD, stroke or death, or end of follow up. Time to event was calculated in years = Time of event (admission date) minus start of follow-up for those who had an event, and end of follow-up minus start of follow-up for those who did not have event. SPSS versions 19.11 and SAS 9.3 were used for the statistical analyses.

## Results

A description of the study population overall and stratified according to tertiles of neuroticism can be seen in Table 1. The prevalence of depression was 14.4% (*n* = 1488). Mean t-score for neuroticism was 145 (SD 24.0); somatic trait anxiety 47.9 (SD 9.7), stress susceptibility 51.1 (SD 8.1) and for embitterment 46.5 (SD 9.9) respectively. High level of neuroticism was present in 27.7% (*n* = 2866) of the participants and of these persons 65.0% (*n* = 1862) were not depressed and 35.0% (*n* = 1004) were depressed. During the follow-up period, 5.1% (*n* = 537) experienced CVD, 3.1% (*n* = 325) with ischemic/hypertensive heart disease and 2.4% (*n* = 248) strokes. Depression was associated with an increased risk for CVD (OR 1.9 (95%CI 1.5,2.4) (as published elsewhere) [20]. Having a high level of neuroticism was also associated with an increased risk for CVD; OR 1.2 (95%CI 1.1, 1.3).

**Table 1:** Characteristics of the population, overall and stratified according to levels^a^ of neuroticism

	Overall	Neuroticism			
		Lowest tertile	Middle tertile	Highest tertile	*P* value *
	*N* = 10,341	*n* = 2828	*n* = 2798	*n* = 2866	
	n (%)	n (%)	n (%)	n (%)	
Age groups					
30–45 years	2877 (33.9)	824 (9.7)	1041 (12.3)	1012 (11.9)	
46–60 years	2740 (32.3)	958 (11.3)	849 (10.0)	933 (11.0)	
61–70 years	2112 (24.9)	750 (8.8)	654 (7.7)	708 (8.3)	
>71 years	758 (8.9)	294 (3.5)	251 (3.0)	213 (2.5)	<0.001
Male gender	3593 (42.3)	1347 (15.9)	1114 (13.1)	1132 (13.3)	<0.001
Socio economic position					
High and intermediate level salary	3713 (54.6)	1258 (18.5)	1244 (18.3)	1211 (17.8)	
Assistant –non manual workers	1181 (17.4)	39 2(5.8)	354 (5.2)	435 (6.4)	
Skilled workers	511 (7.5)	177 (2.6)	189 (2.8)	145 (2.1)	
Unskilled and semiskilled workers	827 (12.2)	283 (4.2)	263 (3.9)	281 (4.1)	
Self-employed (other than professional)	569 (8.4)	183 (2.7)	184 (2.7)	202 (3.0)	0.03
Prevalent IHD or stroke at baseline	267 (2.6)	46 (0.5)	68 (0.8)	96 (1.1)	<0.001
Prevalent hypertension at baseline	600 (7.1)	188(2.2)	174(2.0)	238 (2.8)	0.005
Prevalent diabetes mellitus at baseline	171 (2.1)	47(0.6)	40(0.5)	84 (1.0)	<0.001
Smoking					
Regular smoker	1282 (15.2)	360 (4.3)	381 (4.5)	541 (6.4)	
Occasional smoker	888(10.5)	275 (3.3)	284 (3.4)	329 (3.9)	
Ex-smoker	2497 (29.5)	807 (9.5)	815 (9.6)	875 (10.4)	
Never smoker	3786 (44.8)	1367 (16.2)	1309 (15.5)	1110 (13.1)	<0.001
Physical activity^b^	4565 (53.8)	1643 (19.4)	1547 (18.29)	1375 (16.2)	<0.001
Mean BMI (SD) in kg/m2	24.97 (3.9)	24.9 (3.6)	24.9 (3.7)	25.3 (4.2)	0.001
Hazardous alcohol use	2163 (25.5)	467 (5.5)	704 (8.3)	992 (11.7)	<0.001
Depression	1242 (14.6)	40 (0.5)	198 (2.3)	1004 (11.8)	<0.001

* Physical activity is defined as habitual physical activity at least 3 times a week

^a^ Tertiles were taken at 33.3 and 66.6%

^b^ Chi square test was used to calculate *p* values

The analyses of multiplicative interaction between depression and neuroticism for future risk of CVD, hypertensive/Ischemic heart disease and stroke resulted in *p*-values for the Log likelihood ratios of 0.4, 0.4 and 0.6 respectively indicating no multiplicative interaction. Additive interaction is demonstrated in Table 2 showing Hazard ratios and Synergy Index (95% CI) between Neuroticism and Depression for CVD, IHD and Stroke. For CVD, those who were depressed, the HR (95%CI) ranged from 1.0 (95%, CI 0.5, 2.0) to 2.0 (95%, CI 1.5, 2.6) for low and high level neuroticism respectively in analyses adjusted for age and gender. The corresponding synergy index is 1.7 (95% CI, 0.4, and 6.3). Similar risk estimates and synergy index for outcome of IHD and Stroke are shown.

**Table 2:** Hazard Ratio and Synergy index between Neuroticism and Depression for CVD, IHD and Stroke

	CVD^a^		Hypertensive /Ischemic heart disease^a^		Stroke^a^	
	*N* = 537		*N* = 325		*N* = 248	
	Level of Neuroticism^b^		Level of Neuroticism^b^		Level of Neuroticism^b^	
	Low	High	Low	High	Low	High
	HR (95%CI)	HR (95%CI)	HR (95%CI)	HR (95%CI)	HR (95%CI)	HR (95%CI)
Depression						
No	1 (Ref)	1.4 (1.1,1.7)	1 (Ref)	1.7 (1.3,2.2)	1 (Ref)	1.2 (0.8,1.6)
Yes	1.0(0.5,2.0)	2.0 (1.5,2.6)	1.0 (0.4,2.8)	2.1 (1.5,3.0)	1.3 (0.5,3.1)	2.0 (1.3,2.9)
Synergy index (95%CI)		1.7(0.4,6.3)		1.3(0.3,5.6)		1.7 (0.2,12.1)

^a^ adjusted for age and gender

^b^ Low level neuroticism is categorized based on the lower and middle tertile

Table 3 demonstrates Hazard ratios for CVD, according to depression status and neuroticism after excluding those who had baseline CVD. For CVD, those who were depressed, the HR (95%CI) ranged from 1.0 (95%, CI 0.4, 2.3) to 1.9 (95%, CI 1.3, 2.6) for low and high level neuroticism respectively in analyses adjusted for age and gender, socioeconomic position and history diabetes and hypertension. The association remained after additional adjustment for lifestyle risk factors. Further subgroup analysis for hypertensive/ischemic heart disease and stroke demonstrated similar increased hazard ratios.

**Table 3:** Hazard Ratio for CVD, IHD and Stroke according to depression status and level of neuroticism, Adjusted Analysis

	CVD				Hypertensive/Ischemic heart disease				Stroke			
	*N* = 537				*N* = 325				*N* = 248			
	Level of neuroticism		Level of neuroticism		Level of neuroticism		Level of neuroticism		Level of neuroticism		Level of neuroticism	
	Lower	High	Lower	High	Lower	High	Lower	High	Lower	High	Lower	High
	Model 1^a^		Model 2^b^		Model 1^a^		Model 2^b^		Model 1^a^		Model 2^b^	
Depression	HR(95% CI)	HR(95% CI)	HR(95% CI)	HR(95% CI)	HR(95% CI)	HR(95% CI)	HR(95% CI)	HR(95% CI)	HR(95% CI)	HR(95% CI)	HR(95% CI)	HR(95% CI)
No	1 (Ref)	1.4 (1.1,1.8)	1 (Ref)	1.4 (1.1,1.8)	1 (Ref)	1.7 (1.3,2.4)	1 (Ref)	1.7 (1.2,2.3)	1 (Ref)	1.4(1.0,2.0)	1(Ref)	1.3 (1.0,2.0)
Yes	1.0 (0.4,2.3)	1.9 (1.3,2.6)	1.0 (0.4,2.3)	1.8 (1.2,2.4)	1.3 (0.5,3.5)	2.0 (1.3,3.0)	1.2 (0.4,3.2)	1.8 (1.2,2.8)	1.3 (0.5,3.5)	2.0 (1.3,3.0)	1.4(0.5,3.7)	1.9 (1.2,3.0)

^a^Adjusted for age and gender, SEP, prevalent hypertension, diabetes

^b^ Adjusted for age, gender, SEP, prevalent hypertension, diabetes, physical activity, hazardous alcohol intake, BMI and smoking

## Discussion

In this large population-based cohort of 10,341 adults, we found associations between depression and high levels of neuroticism independently for future risk of CVD. These results are in agreement with previous studies [16, 50, 51]. Further, our study found evidence of a synergistic interaction between neuroticism and depression on future risk for CVD. This implies that high level of neuroticism increases the risk of CVD in depressed patients.

Previous studies have concluded that high neuroticism is an important predictor of depression after stroke [17, 52]. Distressed personality is a predictor of adverse cardiac outcomes after acute MI, irrespective of disease severity and the presence of depression but the results have not been consistent [53–55]. So far there is only one study, a 9-year follow-up study of 2050 participants, Longitudinal Aging Study Amsterdam (LASA) by Marijnissen et al., which has discussed the effect of neuroticism on the relation between depression and future risk of stroke. The study concluded that older persons with depression and low level of neuroticism had a higher risk of developing stroke, thus indicating a negative interaction between depression and neuroticism in predicting future risk of CVD [19]. In contrast, our study demonstrates that there is an interaction between neuroticism and depression in predicting risk for CVD. The reasons for this could be the relatively reduced power of the former study due to selective dropout of persons with missing neuroticism scores in depressed individuals. Moreover, the cardiovascular outcomes in LASA were based on self-reports, medication or GP based diagnoses. This might have resulted in misclassification bias, as many medications that are used for stroke and myocardial infarctions are also used for hypertension. Thirdly, the number of participants with stroke within subgroups was rather low, especially in the subgroup of non-depressed, non-cardiac patients. Our findings that persons affected by depression who have high levels of neuroticism are at higher risk for CVD, emphasize the need for additional treatment besides antidepressant drugs. In persons scoring higher on neuroticism initial treatment with pharmacotherapy might be necessary since it may target neural systems involved in dysregulated emotion. This could then be followed by cognitive behavioral therapy (CBT) which is a well-documented treatment for both depression and neuroticism [31] and have a protective effect on depression recurrence [56].

The evidence for the underlying mechanism between neuroticism and depression leading to CVD has been highlighted in a few studies. Studies report that people scoring high on neuroticism and having depression might have less vascular risk factors (hypertension, diabetes mellitus, hypercholesterolemia, smoking, obesity and low physical activity) for stroke, while those scoring low on neuroticism might have more vascular risk factors as a potential explanation for the increased risk of future stroke [57, 58]. In contrast we suggest, based on the results from this study, that since both depression and neuroticism are independent risk factors for CVD, the vascular risk factors linked to these risk factors are shared and hence leads to increased CVD. These vascular risk factors include hypertension, diabetes, smoking and socioeconomic status [9, 59]. Heart rate variability is another biomarker for neuroticism, which partly accounts for its phenotypic association with CVD, and depression. It is associated with neuroticism, depression and CVD; however the path between heart rate variability and depression is not causal [60].

The strengths of this study are the longitudinal design, the use of a population-based sample and the validated instruments for assessing depression and neuroticism. We also excluded participants with CVD at baseline to prevent misclassification of outcome. Our study also has some limitations that have to be acknowledged. The low response rates in W1 (53%) and slightly low baseline prevalence of depression is a limitation [23]. Since persons severely affected by depression most likely were non responders and this might have underestimated the results. Those who had received in-patient psychiatric care were less likely to participate, which potentially limits the generalizability. However, the non-participation analysis revealed that the odds ratios for associations between depression and gender, income, country of origin and education were similar among participants and non-participants [23, 24]. There might have been some change in the degree of neuroticism over a 10-year follow-up and this might have resulted in slight changes in the risk estimates. As the follow-up period started from 2001, some participants might not have had sufficient latent time for the outcome to occur. We were not able to show multiplicative interaction in this study, however we showed additive interaction which has more public health significance and It has been argued that the assessment of interaction on the additive scale is more indicative of the underlying causal mechanism [48]. Although this study was able to adjust for common mediators and confounders, it did not offer the possibility to adjust for dyslipidemia, blood glucose, type and use of antidepressants and treatment compliance due to lack of data.

## Conclusion

Higher levels of neuroticism increased the risk of CVD in depressed persons. We found a synergistic interaction between neuroticism and depression in predicting future risk of CVD. Further research is required to test early therapeutic interventions for concomitant neuroticism and depression and its effect on CVD.

## Appendix

**Table 4:** Personality scales reflecting neuroticism; mean and t score

Scale	Question number in SSP	Question	Mean (SD)score of individual questions^a^	Mean(SD) score of scale	Mean (SD)t score of scale ^c^
Somatic Trait Anxiety (STA)					
	1	Quite often, I found myself without reason clenching my jaw.	1.8 (0.97)	1.7 (0.5)	47.9 (9.7)
	14	I often feel restless, as if I wanted something without knowing what.	2.0 (0.9)		
	27	I often feel stiff and tense the body	2.0 (0.9)		
	40	Sometimes, my heart thumps hard or beat irregularly without tangible reason.	1.5 (0.8)		
	53	I can suddenly start sweating for no particular reason.	1.5 (0.8)		
	66	I jerk violently to unexpected sounds.	1.9 (0.9)		
	79	Sometimes I get a feeling of not getting enough air to breathe.	1.4 (0.8)		
Stress Susceptibility(SS)					
	3	I can too easily get tired and stressed.	2.2 (0.8)	2.0 (0.4)	51.0 (8.1)
	−16^b^	I can easily be disturbed when I’m doing a job.	2.0 (0.7)		
	29	In order to get anything done, I have to consume more power than most.	1.5 (0.7)		
	−42	I tend to concentrate even if the surroundings are distracting	2.2 (0.7)		
	55	I get easily stressed when I am asked to speed up my work.	2.0 (0.8)		
	−68	I feel calm and confident even if I have to face new challenges.	2.2 (0.7)		
	81	I find that I have less energy than most of my acquaintances.	1.6 (0.8)		
Embitterment(E)					
	9	I have had it quite difficult in life	1.8 (0.8)	1.6 (0.4)	46.5 (9.9)
	22	I never seem to be able to avoid getting into jams.	0.6 (1.3)		
	35	I have often got into trouble, even though it was not my fault.	1.3 (0.6)		
	48	It looks as if I would never get any chance to get anywhere in life.	1.5 (0.7)		
	61	I seem to more often than others do things that I later regret	1.4 (0.6)		
	74	It happened that I envied people who have been lucky in life	2.1 (0.9)		
	87	I feel often like I did something bad or wrong.	1.5 (0.7)		

^a^ Each item is given as a statement with a four-point response format, ranging from 1 to 4, 1 point for the “disagree” and so on to 4 points for the “agree”

^b^ Minus in front of the question number means the scoring was reversed. This applies to the item 7, 16, 30, 38, 42, 68, 85, and 86

^c^ t scores have been calculated according to Gustavsson, J.P., et al., Swedish universities Scales of Personality (SSP): construction, internal consistency and normative data. Acta Psychiatr Scand, 2000: 102(3)

## Abbreviation

CVD: Cardiovascular diseases
HTN: Hypertension
IHD: Ischemic heart diseases
MDI: Major depression inventory
SSP: Swedish scale of personality
